# Polysaccharides-Rich Extract of *Ganoderma lucidum* (M.A. Curtis:Fr.) P. Karst Accelerates Wound Healing in Streptozotocin-Induced Diabetic Rats

**DOI:** 10.1155/2013/671252

**Published:** 2013-11-24

**Authors:** Poh-Guat Cheng, Chia-Wei Phan, Vikineswary Sabaratnam, Noorlidah Abdullah, Mahmood Ameen Abdulla, Umah Rani Kuppusamy

**Affiliations:** ^1^Mushroom Research Centre, University of Malaya, 50603 Kuala Lumpur, Malaysia; ^2^Institute of Biological Sciences, Faculty of Science, University of Malaya, 50603 Kuala Lumpur, Malaysia; ^3^Department of Biomedical Science, Faculty of Medicine, University of Malaya, 50603 Kuala Lumpur, Malaysia

## Abstract

*Ganoderma lucidum* (M.A. Curtis:Fr.) P. Karst is a popular medicinal mushroom. Scientific reports had shown that the wound healing effects of *G. lucidum* were partly attributed to its rich polysaccharides. However, little attention has been paid to its potential effects on wounds associated with diabetes mellitus. In this study, we evaluated the wound healing activity of the hot aqueous extract of *G. lucidum* in streptozotocin-induced diabetic rats. The extract of *G. lucidum* was standardised based on chemical contents (w/w) of total polysaccharides (25.1%), ganoderic acid A (0.45%), and adenosine (0.069%). Six groups of six rats were experimentally wounded in the posterior neck region. Intrasite gel was used as a positive control and aqueous cream as the placebo. Topical application with 10% (w/w) of mushroom extract-incorporated aqueous cream was more effective than that with Intrasite gel in terms of wound closure. The antioxidant activity in serum of rats treated with aqueous extract of *G. lucidum* was significantly higher; whereas the oxidative protein products and lipid damage were lower when compared to those of the controls. These findings strongly support the beneficial effects of standardised aqueous extract of *G. lucidum* in accelerating wound healing in streptozotocin-induced diabetic rats.

## 1. Introduction

Diabetes mellitus is a metabolic disorder characterised by hyperglycemia with impaired carbohydrate, fat, and protein metabolism. The diabetic condition can be due to defects in insulin secretion, action, or both [1]. It affects more than 180 million individuals worldwide and by 2030 these numbers are projected to double [2]. Diabetes mellitus has led to debilitating consequences such as vasculopathy, retinopathy, and neuropathy [1]. More than 80% of diabetes mellitus is Type 2 diabetes (noninsulin dependent) characterised by peripheral resistance to the action of insulin and decreased peripheral glucose uptake or increased hepatic glucose output [1].

According to the statistics provided by the National Diabetes Information Clearinghouse, 15% of diabetic individuals suffered from diabetic foot ulcers that caused lower limbs to be amputated [3]. The hyperglycemic state in diabetic patients, especially those with peripheral vasculopathy interrupts proper wound healing. Wound healing occurs as a cellular response to injury and involves activation of keratinocytes, fibroblasts, endothelial cells, macrophages, and platelets. Many growth factors and cytokines released by these cell types are needed to coordinate and maintain healing [1, 4]. Furthermore, poor blood circulation and the oxygen supply in the affected area could lead to infection and gangrene formation [5]. This often leads to increased morbidity and mortality. Despite the existence of protocols to standardise wound care, the physiological impairments that can result in a diabetic foot ulcer (DFU) further complicate the healing process [6]. Currently, the wounds in diabetic patients are managed with antiseptic or antibiotic creams or gels such as Intrasite gel for wound dressing. Wound healing takes prolonged periods and the healed wounds leave scars. In the search and discovery of safe and effective wound healing agents, natural products from plant and mushrooms are currently being explored.

Mushrooms have enormous potential as a source of both dietary protein and health-enhancing dietary supplements [7]. Among the medicinal mushrooms, *Ganoderma *species are much sought after for their wide array of medicinal properties. *Ganoderma lucidum *(M.A. Curtis:Fr.) P. Karst which belongs to the Polyporaceae family has long been known and used extensively in traditional Chinese medicine. In Malaysia, due to the high humidity and temperature throughout the year this mushroom is widely cultivated. Chemical analysis of the fruiting bodies of *G. lucidum* showed the presence of polysaccharides and triterpenoids [8–10] that may have therapeutic values in the treatment or prevention of peripheral or central inflammatory diseases. *Ganoderma *spp. are a natural source of potent bioactive antioxidant metabolites [11]. Total phenols were the major naturally occurring antioxidant components found in both *G. lucidum *and* G. tsugae* [12]. It has been reported that some plants, for example, *Annona squamosa* L. (Annonaceae), commonly known as the custard apple [13], as well as *Catharanthus roseus* L (Apocynaceae) flower [14], promoted wound healing in diabetic rats via free radical scavenging activity of flavonoids. To date, reports of the applications of the medicinal properties of mushrooms in the healing of wounds in diabetic rats are rather rare. Kwon et al. [15] reported that the cauliflower mushroom, *Sparassis crispa* Wulf.:Fr. (Aphyllophoromycetideae), improved the healing of diabetic wounds. The high *β*-glucan (more than 40%) accounted for the increase in the migration of macrophages and fibroblasts as well as elevated collagen synthesis. Sacchachitin and chitin membrane prepared from the aqueous extracts of *G. tsugae* were also found to have wound healing properties [16, 17]. 

In our preliminary study using normal rats without diabetes, the period of reepithelialisation and wound closure showed no significant difference (*P* > 0.05) between the Intrasite-gel- and *G. lucidum*-treated groups (unpublished data). The effect of wound healing by the hot aqueous extract of *G. lucidum* was comparable to that by Intrasite gel and this might be due to its high content of polysaccharides (25.1%) and the synergistic reactions combining all the medicinal properties as a whole. As wound healing in hyperglycemic state is difficult and challenging, the aim of this study was to further investigate the effect of the hot aqueous extract of *G. lucidum *on wound healing and the oxidative damage in streptozotocin-induced diabetic rats.

## 2. Materials and Methods

### 2.1. Preparation and Standardisation of Hot Aqueous Extract of *G. lucidum*

The fresh fruiting bodies of *G. lucidum *were obtained from Ganofarm Sendirian Berhad, a mushroom farm in Tanjung Sepat, Selangor, Malaysia. The production of *G. lucidum *was reported previously [18, 19]. The powdered fruiting bodies were subjected to hot water extraction (5 : 200, w/v) at 100°C for 8 hours. The resulting aqueous extract was freeze-dried and kept at −20°C prior to use. Heavy metal composition and microbial load of selected pathogens were analyzed by Nova Laboratory, Sepang, Malaysia, using proprietary methodology. A voucher specimen of *G. lucidum *(KLU-M 1233) was deposited in the herbarium of Mushroom Research Centre, University of Malaya.

### 2.2. Determination of Total Polysaccharides Content in Hot Aqueous Extracts of *G. lucidum*

The total polysaccharide content of the hot aqueous extract was determined using the phenol-sulphuric acid method with d-glucose as in [20]. Briefly, one mL of 5% (w/v) phenol was added to one mL of sample solution, followed by five mL of concentrated H_2_SO_4_. The absorbance was measured using a spectrophotometer (Shimadzu series 1601 UV/Vis) after 10 minutes at 483 nm. 

### 2.3. Quantification of Ganoderic Acid and Adenosine Using HPLC

For determination of ganoderic acid A, Perkin Elmer Series 200 liquid chromatography equipped with a Perkin Elmer Series 200 UV detector was used. The detector signal was recorded by the Turbochrom workstation software. The column was Hypersil BDS C18 (4.6 × 250 mm) with Alltech refillable C18 Guard column (10 × 4.6 mm) (Alltech, USA). The mobile phase consisted of 5% (v/v) acetic acid in methanol and the flow rate was 1.0 mL/min. The calibration curve was prepared by injecting a series of ganoderic acid A (Sigma) reference standard dilutions. Quantification and validation of adenosine were also performed in Perkin Elmer Series 200 liquid chromatography as mentioned. The mobile phase was methanol: 10 mM monobasic potassium phosphate (15 : 85), pH 5.0, and the flow rate was 1.5 mL/min. Both ganoderic acid A and adenosine were quantified by means of calibration curves obtained from commercial standards of these compounds (Sigma).

### 2.4. Determination of Cupric Reducing Antioxidant Capacity (CUPRAC) of Hot Aqueous Extract of *G. lucidum*

The cupric reducing antioxidant capacity (CUPRAC) of the hot aqueous extract of *G. lucidum* was determined according to the method of Apak et al. [21]. Briefly, to a mixture of one mL of copper(II) (10^−2^ M), neocuproine (7.5 × 10^−3^ M), and ammonium acetate buffer solution (1 M), freshly-prepared mushroom extracts of varying concentrations were added to make up a final volume of four mL. After incubation at room temperature (25 ± 2°C) for 30 minutes, the absorbance at 450 nm was recorded against a reagent blank. The results of antioxidant activity were expressed in absorbance at 450 nm and compared with ascorbic acid as a positive control.

### 2.5. Preparation of Mushroom Extract-Incorporated Treatment Cream

The hot aqueous extract at concentrations of 5%, 10%, 15%, and 20% (w/w) was mixed with aqueous cream homogeneously. Aqueous cream was obtained from the Department of Pharmacy, Faculty of Medicine, University of Malaya (a product of Sunward Pharmaceutical Sendirian Berhad, MAL 19920890 X). 

### 2.6. Experimental Animals

Healthy adult male Sprague Dawley rats were obtained from the animal house, Faculty of Medicine, University of Malaya. The rats were divided randomly into six groups of six rats each. Each rat weighed between 180 to 250 g and was housed separately (one rat per cage). The animals were maintained on a standard pellet diet and tap water. The study conformed to the Principles of Laboratory Animal Care and was approved by the Ethics Committee of University of Malaya with the Ethic number ISB/14/10/2009/CPG (R). All animals received care according to the criteria outlined in the “Guide for the Care and Use of Laboratory Animals” prepared by the National Academy of Sciences and published by the National Institutes of Health.

### 2.7. Diabetes Induction

Streptozotocin (STZ) was purchased from Sigma-Aldrich (St. Louis, MO, USA). After an overnight fast, diabetes mellitus was induced in six groups of rats by a single intraperitoneal injection of streptozotocin (STZ; 45 mg/kg) dissolved in citrate buffer (0.1 M, pH 4.0). Blood was drawn from the tail vein on the 7th day after the STZ injection, and the fasting blood glucose levels were estimated using a glucometer (Ames, Bayer Diagnostic). Rats with fasting blood glucose levels higher than 10 mmol/L (or 200 mg/dL) were considered diabetic and were included for the experiment [22].

### 2.8. Excision Wound Creation

Excision wounds were created on the 7th day after the induction of diabetes in all rats. The animals were anaesthetised with 2 mL of diethyl ether (Sigma, 98% purity). The skin was shaved using an electric clipper, disinfected with 70% alcohol, and 0.5 mL of lignocaine HCl (2%, 20 mg/mL) was injected as a local anaesthetic agent. The area of wound was outlined with methylene blue using a circular stencil. A full thickness of the excision wound of 2.0 cm in length and 0.2 cm depth was created as described by Nayak and Pinto Pereira [14]. Care was taken to avoid injuring the muscle layer, and the tension of skin was kept constant during the procedure. The wound areas were measured using a graph paper.

### 2.9. Topical Application of Treatment Creams

The wounds of Group 1 rats were dressed with a thin layer of aqueous cream as a blank placebo twice daily. The wounds of Group 2 rats were dressed topically twice daily with 0.2 mL of Intrasite gel as the positive control. Intrasite gel, which is a trademark of Smith and Nephew Ltd., was purchased from the University Malaya Medical Centre Pharmacy. Rats in Group 3, 4, 5, and 6 rats were treated with a thin layer of aqueous cream containing 5%, 10%, 15%, and 20% (w/w) aqueous extracts of *G. lucidum*, respectively. All applications of cream were performed with appropriate care twice a day.

### 2.10. Determination of the Period of Reepithelialisation

The wounds of all animals under the different treatments were observed daily. The period of reepithelisation was assessed by counting the number of days required for the complete healing including eschar falloff without any residual raw wound [13].

### 2.11. Determination of the Wound Closure

The wound area (mm^2^) was measured at 0, 1, 4, 8, 12, and 16 days after wounding using transparency paper and a permanent marker. The rate of wound closure which is expressed as the percentage of wound reduction from the original wound was calculated using the following formula [14]:

(1) Percentage of wound closure (%)=[(wound area on⁡ day 0−wound area on⁡     postoperation day)  ×(wound  area  on⁡  day  0)−1]×100%.

### 2.12. Histological Evaluation of Healed Wounds

The specimens of skin from healed wounds and surrounding tissues were excised and stained for histological studies. Three sections (5 **μ**m thickness) from each rat were prepared for hematoxylin and eosin (H&E) and Masson Trichrome Staining, and stained sections of each wound were examined by light microscopy. Scar width (mm) which is the junction gap between the normal dermis and dermis in the wound tissues was measured [23]. The morphological changes (fibroblast, inflammatory cell, neovascularisation, and collagen) were recorded. 

### 2.13. Determination of *In Vivo* Antioxidant Activity of Experimental Rats

Blood sample was collected from the experimental rats on postoperation days 7 and 16. Antioxidant activity in the blood serum was determined by the CUPRAC method [21], which utilises copper(II)-neocuproine reagent as the chromogenic oxidising agent. Briefly, the mixture of 1 mL of CuCl_2_ (10^−2^ M), neocuproine (7.5 × 10^−3^ M), and ammonium acetate buffer solution (1 M) were added into a cuvette. Then, 1090 *μ*L of distilled water with 10 *μ*L of blood serum was added into the reagent mixture and incubated at room temperature (25 ± 2°C) for 30 minutes. The absorbance at 450 nm was recorded against a reagent blank. The result of antioxidant activity was expressed as absorbance at 450 nm against blank. Ascorbic acid was used as positive control. Each sample of serum was triplicated for absorbance reading, *n* = 6 for each group of experimental animals.

### 2.14. Assessment of Oxidative Damage

#### 2.14.1. Advanced Oxidation Protein Product (AOPP) Assay

Advanced oxidation protein product (AOPP) was determined by the method of Witko-Sarsat et al. [24]. Briefly, AOPP was determined spectrophotometrically using a microplate reader and was calibrated with chloramine-T solutions. Reagent mixture was prepared by adding 81 mL of PBS solution, 15 mL of acetic acid (50%), and 4 mL of potassium iodide. Then, 18 *μ*L of plasma sample was added to 200 *μ*L of reagent mixture in a 96-well microplate reader. The absorbance at 340 nm was recorded against a reagent blank. AOPP assay for each plasma sample was carried out in triplicates, *n* = 6 for each group of experimental animals. AOPPs were expressed as *μ*mol/L chloramine-T equivalents. 

#### 2.14.2. Lipid Hydroperoxide (LPO) Assay

Lipid hydroperoxide (LPO) was determined according to the method of Esterbauer and Cheeseman [25]. Malondialdehyde (MDA) was assayed as a marker of lipid peroxidation using colorimetric reaction, which uses 1-methyl-2-phenylindole (MPI) as chromogen. Condensation of one molecule of MDA with two molecules of MPI under acidic condition results in the formation of a chromophore which has maximum absorbance at 586 nm. A total of 150 *μ*L of serum sample was added to 375 *μ*L of MPI (10.3 mM) in acetonitrile and 225 *μ*L HCl (5 M). The mixture was incubated in a water bath at 45°C for 40 minutes. Tetraethoxypropane (TEP) was used as standard solution. After centrifugation at 10,000 ×g for 5 minutes, 200 *μ*L of the reaction mixture was pipetted into a 96-well plate and read at 586 nm in an ELISA reader (UV 1601 spectrophotometer, Shimadzu, Japan). Concentration of MDA was expressed as nM of TEP equivalents. Each of the serum samples was assayed in triplicates, *n* = 6 for each group of experimental animals.

### 2.15. Statistical Analysis

All values are reported as mean ± standard error mean (S.E.M). Statistical evaluation of the data was done by one way analysis of variance (ANOVA) followed by Bonferroni's multiple comparison tests. Differences were considered statistically significant when *P* < 0.05.

## 3. Results

### 3.1. Standardisation and Analyses of Aqueous Extract of *G. lucidum*

**Figure 1:**
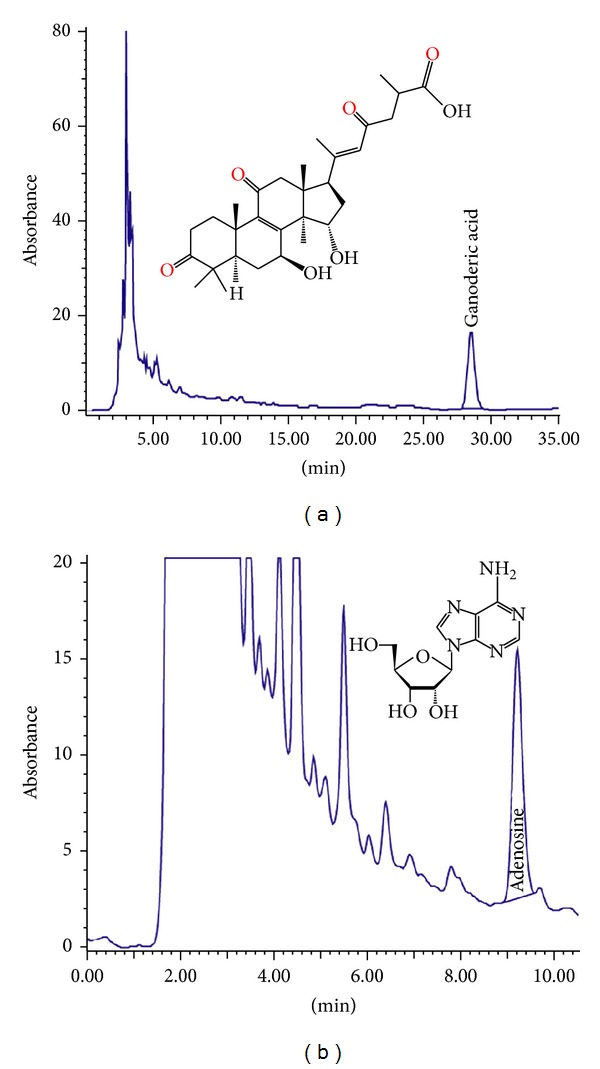
Chromatograms of ganoderic acid A (a) and adenosine (b) from the hot aqueous extract of *Ganoderma lucidum*.

The extraction yield of aqueous extract from the fruiting bodies of *G. lucidum* was 8.98% (w/w) (Table 1). The high performance liquid chromatography (HPLC) revealed that the aqueous extract contained 0.45% and 0.07% (w/w) of ganoderic acid A (Figure 1(a)) and adenosine (Figure 1(b)), respectively. Total polysaccharides were found to be 25.1% by using the phenol-sulphuric acid method. The heavy metal content was determined by using atomic absorption spectrophotometer. Analysis showed that the extract contained <5.0 ppm of arsenic, <10.0 ppm of lead, <0.5 ppm of mercury, and <0.3 ppm of cadmium. The microbial tests showed that the total bacterial count, yeast and mould count, and enterobacteriaceae were not more than 10^5^, 10^4^, and 10^5^ cfu/g, respectively. *Salmonella *spp., *Escherichia coli, Staphylococcus aureus,* and *Pseudomonas aeruginosa* were not detected in the aqueous extract of *G. lucidum* (Table 1).

### 3.2. *In Vitro* Antioxidant Activities of Aqueous Extract of *G. lucidum*

Cupric reducing antioxidant capacity (CUPRAC) assay was performed in order to have a better understanding of the antioxidative characteristic of the standardised aqueous extract of *G. lucidum*. The assay was based on the measurement of absorbance at 450 nm by the formation of a stable complex between neocuproine and copper(I). The antioxidant activity increased with increasing concentration of the extracts (Table 2). There was no significant difference (*P* > 0.05) in the activity of the aqueous extract of *G. lucidum* at 1.5 mg/mL compared to ascorbic acid (5 × 10^−3^ g/mL).

### 3.3. Wound Healing Activity of Treatment Creams on Excision Wounds in Diabetic Rats

#### 3.3.1. Wound Reepithelialisation

**Figure 2:**
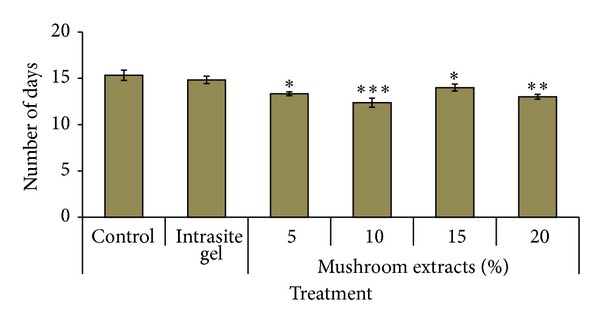
Time required for wound reepithelialisation in diabetic rats by aqueous extract of *G. lucidum*. Values are expressed as mean ± S.E.M. *P* value less than 0.05 is considered as significant difference. *P < 0.05; **P < 0.01, and ***P < 0.001 comparison between mushroom extract-treated and control rats.

Wounds dressed with mushroom extracts as well as Intrasite gel (positive control) showed considerable signs of dermal healing when compared to wounds dressed with aqueous cream only (negative control). Based on Figure 2, the diabetic rats treated with all doses of aqueous extracts of *G. lucidum *healed significantly faster (*P* < 0.05) compared to rats in the negative control group. Notably, treatment with 10% (w/w) of aqueous extract of *G. lucidum * showed the shortest reepithelisation period which was 12.37 ± 0.49 days, then followed by 20%, 5%, and 15% of *Ganoderma* extracts. 

#### 3.3.2. Wound Closure

Table 3 shows the percentage of wound closure at various time intervals. On day 8, contraction of the wound in all the experimental rats was observed. The wound closure for diabetic rats receiving Intrasite-gel treatment at day 8 was 136.86 ± 22.8 mm^2^ (55.0% of wound closure from day 0). It may be due to dehydration of the necrotic tissue and drying of exudates. However, continuous application of the gel caused excessive drying of the wound tissue and wound healing was delayed at a later stage. Interestingly, diabetic rats receiving treatment of 10% (w/w) of *Ganoderma* extract exhibited a significantly (*P* < 0.05) higher wound closure which was 149.12 ± 16.5 mm^2^ (60%) as compared to the Intrasite gel-treated rats on day 8. On day 12, a significant (*P* < 0.05) increase in the percentage of wound closure was noted in all groups. The group treated with 10% (w/w) *Ganoderma* extract showed the highest wound closure which was 242.08 ± 7.8 mm^2^ (97%) and this was significantly higher (*P* < 0.05) than that of Intrasite gel (175.68 ± 23.10 mm^2^; 70%). 

#### 3.3.3. Macroscopic Analysis of Wound

**Figure 3:**
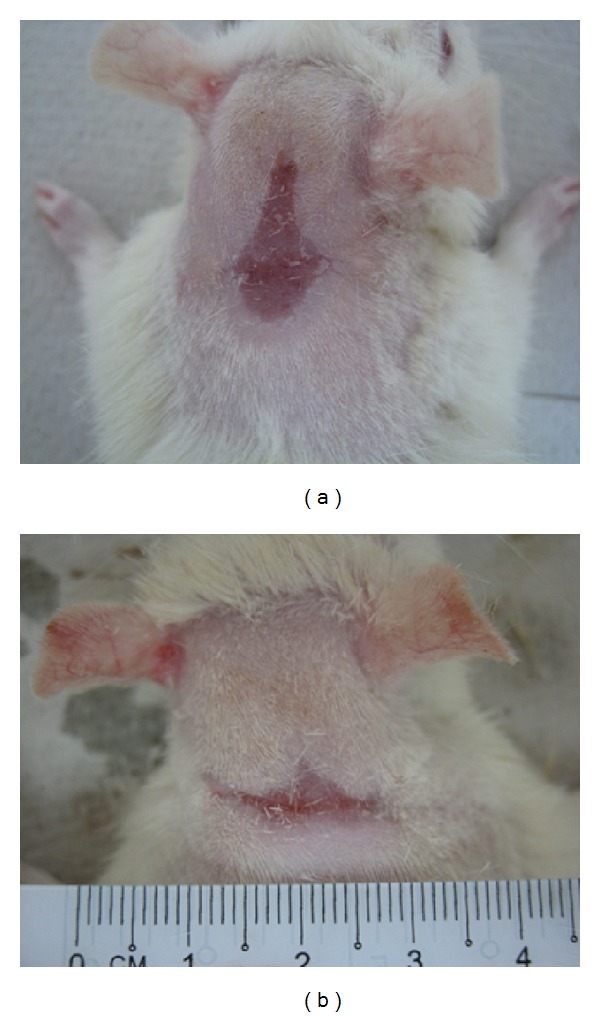
Gross appearance of the healing of excision wound of diabetic rats at day 16 after operation. (a) Wound dressed with placebo cream—control group. (b) Wound dressed with aqueous cream incorporated with 10% (w/w) of hot aqueous extract of *G. lucidum*.

Macroscopic analysis showed that the scar tissue after being treated with aqueous cream (placebo) showed an irregular healing pattern on day 16 after operation (Figure 3(a)). In contrast, the scar tissue of healed wound treated with 10% (w/w) aqueous extract of *G. lucidum *was comparable to a sutured excision wound (Figure 3(b)). This indicated that aqueous extract of *G. lucidum *had stimulated inflammatory cells, fibroblasts, and keratinocytes to the wound site to induce a more rapid maturation of granulation tissue.

#### 3.3.4. Histological Analysis of Wound

**Figure 4:**
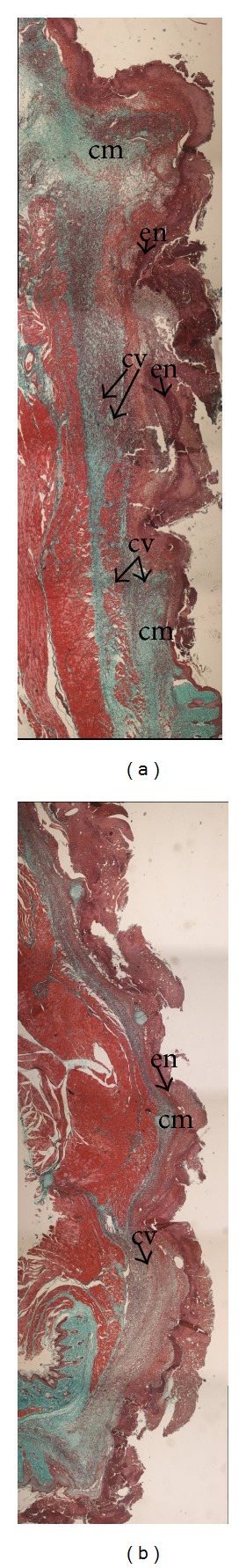
Histological analysis of wound at day 7 after operation (4x Masson Trichome Staining). (a) Wound treated with 10% (w/w) extract of *G. lucidum* showed more collagen matrix with newly formed capillary vessels underneath the endothelial cells. (b) Wound treated with placebo. cm: collagen matrix, cv: capillary vessel, en: endothelial cells.

**Figure 5:**
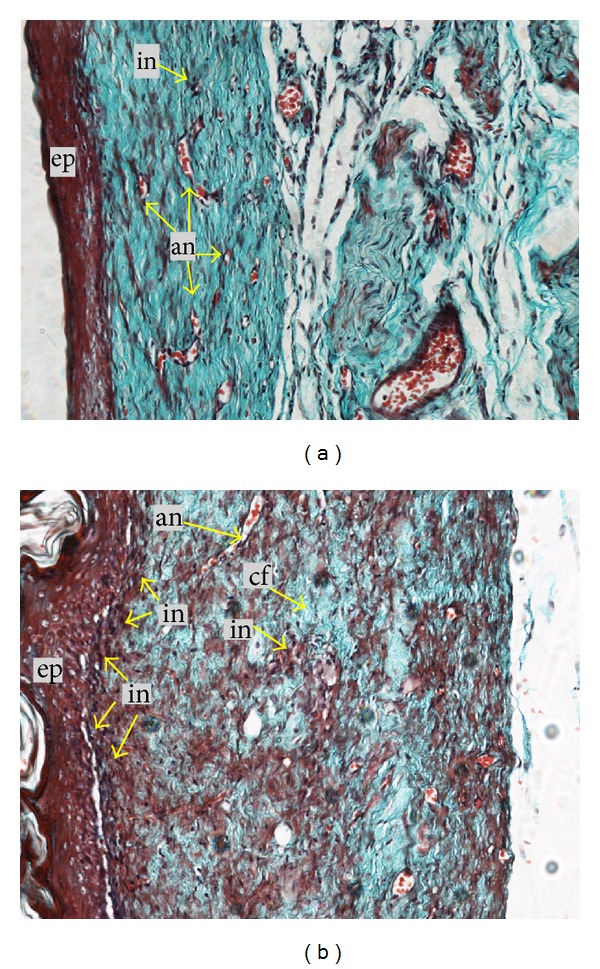
Histological analysis of healed wound at day 16 after operation (20x Masson Trichome Staining). (a) Wound treated with 10% (w/w) extract of *G. lucidum *showed good epithelisation and well-formed granulation tissue, markedly fewer inflammatory cells, and more collagen accompanied with angiogenesis. (b) Wound treated with placebo. ep: epithelialisation, in: inflammatory cell, cf: collagen fibre, an: angiogenesis.

**Figure 6:**
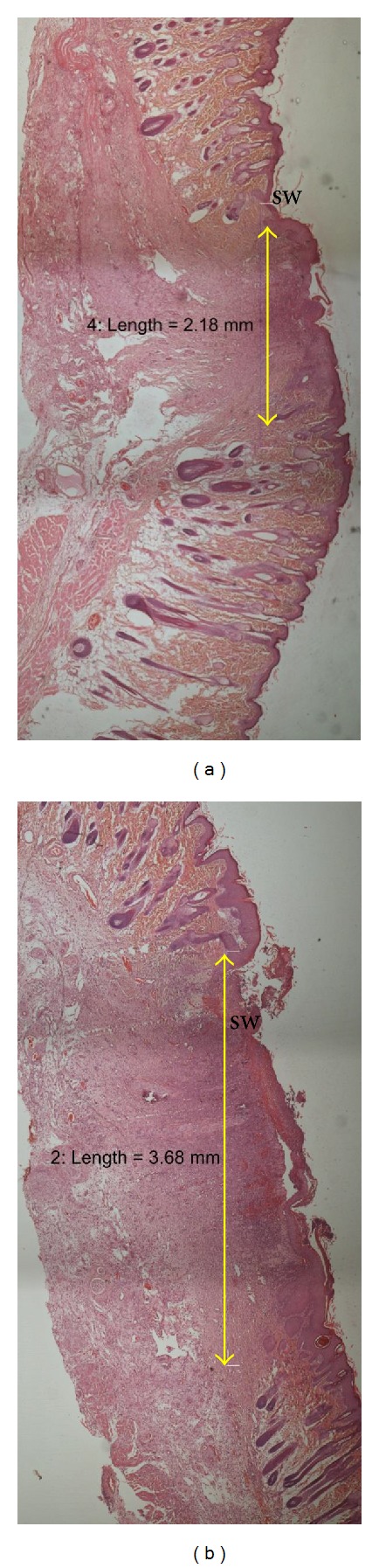
Histological section of healed wound showing the scar-width on day 16 after wounding (H&E, 4x). (a) Wound treated with extract of *G. lucidum*. (b) Wound treated with placebo. sw: scar width.

Histological analysis of wound on day 7 after operation revealed that there was more collagen matrix with newly formed capillary vessels below the endothelial cells in rats treated with extracts of *G. lucidum *(Figure 4(a)) compared to control (Figure 4(b)). This could be due to the upregulation of collagen synthesis and angiogenesis at the wound site, with improved blood circulation which provides more oxygen and nutrients essential for the healing process. Meanwhile, histological analysis of healed wound on day 16 after operation revealed that wounds dressed with 10% (w/w) extract of *G. lucidum *showed good epithelisation and well-formed granulation tissue (Figure 5(a)). They contained markedly fewer inflammatory cells and more collagen accompanied with angiogenesis as compared to control (Figure 5(b)). The scar width was smaller at wound closure in the rats treated with extract of *G. lucidum* (Figure 6(a)) as compared to control (Figure 6(b)). The measurements for the scar width of healed wound were 2.56 ± 0.59 mm, 3.42 ± 0.75 mm, and 2.91 ± 1.06 mm for rats treated with extract of *G. lucidum*, placebo, and Intrasite gel, respectively. Meanwhile the blood vessel count for the three treatment groups was 77 ± 23.65, 68.25 ± 12.58, and 51.40 ± 16.88, respectively.

### 3.4. *In Vivo* Antioxidant Capacity and Oxidative Damage Assessment

*In vivo* antioxidant capacity and oxidative damages during wound healing were quantified in serum of rats on day 7 after operation. Results showed that antioxidant activity using CUPRAC was significantly higher (*P* < 0.05) in the diabetic rats treated with 15% (w/w) aqueous extract of *G. lucidum* when compared to the negative control and Intrasite-gel-treated rats (Table 4). The AOPP levels in diabetic rats treated with 10%, 15%, and 20% (w/w) aqueous extract of *G. lucidum *were significantly reduced (*P* < 0.05). On the other hand, the LPO levels for all the treatments were reduced with no significant difference (*P* > 0.05) to the negative control.

## 4. Discussion

There are three phases in the wound healing process: inflammation, proliferation, and maturation [1, 26]. After initial wounding, blood extravasation causes platelet aggregation and blood clotting. These events initiate inflammation and set the stage for repair processes. During the repair phase, the provisional wound matrix is remodelled and replaced with scar tissue, consisting of new collagen fibres and proteoglycans and elastin fibres, which partially restore the structure and function of the tissue [27]. This is accomplished by the migration, proliferation, and differentiation of epithelial cells, dermal fibroblasts, and vascular endothelial cells from adjacent uninjured tissue to the wound site [28]. Eventually the injured tissue is repaired rather than regenerated [1, 27]. 

Our preliminary study showed that the ethanol extract of *G. lucidum *contained 0.1% of polysaccharides (unpublished data). In this study the hot aqueous extract had higher polysaccharides (25.1%) as compared to the ethanol extract. Therefore the hot aqueous extract was considered rich in polysaccharides; as compared to the ethanol extract. Bae et al. also reported that the polysaccharides isolated from *Phellinus gilvus* (Schw.) Patouillard (mustard-yellow polypore) enhanced dermal wound healing in normal [29] and streptozotocin-induced diabetic rats [30]. These results are in agreement with our study that hot water extraction at 100°C is an appropriate method for polysaccharide extraction in mushroom. Kwon et al. [15] also reported that the *β*-glucan purified from medicinal mushroom* Sparassis crispa* (cauliflower mushroom) increased macrophage infiltration into the wound tissue and enhanced wound healing. Accordingly, the mechanism of *β*-glucan-induced wound healing was associated with increased types I and III collagen biosynthesis. While the *β*-glucan was orally administered to the rats in the study by Kwon et al., in this experiment, the wounds of diabetic rats were treated topically with aqueous cream containing varying concentrations of *G. lucidum* extracts. Most recently, polysaccharides purified from *Tremella fuciformis *(white jelly mushroom) and *Auricularia auricula* (wood ear mushroom) were shown to enhance wound healing using the *ex vivo* porcine skin wound healing model [31]. The water-soluble polysaccharide fractions of *G. lucidum* have been reported to have healing effects especially on ulcer lesions [32, 33]. Elsewhere, Sun et al. [34] reported that *G. lucidum* polysaccharides showed healing effects on intestinal epithelium using a nontransformed small-intestinal epithelial cell line, IEC-6 cells. Despite all the beneficial wound healing effects of *G. lucidum*, little attention has been paid to its effects on wounds associated to diabetes. Further, the major chemical components, ganoderic acid and adenosine, may contribute to the wound healing activities of the hot aqueous extracts of *G. lucidum*.

In this study we used streptozotocin to induce diabetes in rats. Streptozotocin-induced rodent is a model widely used in the study of insulin-dependent diabetes mellitus and hyperglycemia [35]. Through our observations, wound healing in diabetic rats was delayed compared to that in normal healthy rats (unpublished data). The mechanism of wound healing in healthy rats is well guided and it is through integration of multiple signals in the form of cytokines and chemokines released by keratinocytes, fibroblasts, endothelial cells, macrophages, and platelets. However, in diabetic rats, healing impairment is characterised by delayed cellular infiltration and granulation tissue formation, reduced angiogenesis, and decreased collagen and its organisation [36]. The mechanism of this alteration is thought to result from production of high level of reactive oxygen species (ROS) and increased level of apoptosis related to diabetes mellitus, which in turn impairs keratinocyte, endothelial cells, fibroblast, and collagen metabolism. In this study, wound healing on day 12 may be at the most active stage involving inflammation and cell proliferation, where fibroblast proliferated at peak and was responsible for initiating angiogenesis, epithelialisation, and collagen formation. As indicated by the results, aqueous extract of *G. lucidum* stimulated proliferation and migration of fibroblast as well as collagen synthesis in wound healing in diabetic rats.

Low levels of antioxidants accompanied by a slight increase in markers of free radical damage played a significant role in diabetic wound healing [37]. The antioxidant defence system in the body consists of endogenous and exogenous antioxidants that work together at the molecular level to protect cell membrane, lipoproteins, and DNA from the damaging effects of free oxygen radicals [38]. Endogenous antioxidants are enzymes that are primarily physiologic in origin while exogenous antioxidants include nutrients that enter the body through the diet. Aqueous extract of *G. lucidum* which exhibited high antioxidant activity and free radicals scavenging activity may reduce oxidative damage to the cell at wound site. Oxidative damage of proteins is one of the modifications leading to severe failure of biological functions and cell death. Prolonged exposure of protein to reactive molecules leads to spontaneous modifications, such as oxidation to form advanced oxidation protein products (AOPPs) [24]. In our experiment, AOPPs in diabetic rats might be due to infection of wound or stress caused by pathogenesis of diabetes. It appears that antioxidant activity as well as immune modulation of polysaccharides may bring about the wound healing effects of this medicinal mushroom. Nevertheless, the underlying mechanism(s) of *G. lucidum* in wound healing effects in diabetic-induced model need to be further investigated. 

## 5. Conclusion

The present study showed that topical application of aqueous cream incorporated with varying concentrations of the hot aqueous extract of *G. lucidum *significantly (*P* < 0.05) enhanced the rate of wound healing in streptozotocin-induced diabetic rats. The polysaccharide-rich (25.1%, w/w) hot aqueous extract of *G. lucidum*, which also had ganoderic acid and adenosine, increased the *in vivo* antioxidant capacity and reduced the oxidative damage during wound healing in diabetic rats. The mechanisms of wound healing contributed by this medicinal mushroom are yet to be elucidated. Furthermore, identification of other active ingredients, if any, in the hot aqueous extract of *G. lucidum *is warranted. To date, scientific investigations had validated the traditional uses of *G. lucidum *in wound management. Therefore, there is a compelling need to develop a complementary and alternative therapy for impaired wound healing, which may help to avoid amputation and improve quality of life in individuals diagnosed with diabetes. Apart from that,* G. lucidum *can be artificially cultivated and abundantly grown in the tropical country. Therefore, it could be a fairly economical therapeutic agent in diabetic wound management.

## Figures and Tables

**Table 1:** Specifications of standardised aqueous extract of *Ganoderma lucidum*.

Parameters	Specifications
Appearance and colour	Brown-yellow powder
Marker components	
Total polysaccharides	25.1%
Ganoderic acid A	0.45%
Adenosine	0.069%
Microbial tests	
Total bacterial count	Not more than 10^5^ cfu/g
Yeast and mould count	Not more than 10^4^ cfu/g
Enterobacteriacea	Not more than 10^3^ cfu/g
* Salmonella *spp.	Absent
* E. coli *	Absent
* S. aureus *	Absent
* P. aeruginosa *	Absent
Heavy metal tests	
Arsenic	Not more than 5.0 ppm
Lead	Not more than 10.0 ppm
Mercury	Not more than 0.5 ppm
Cadmium	Not more than 0.3 ppm

**Table 2:** Antioxidant activity of standardised aqueous extract of *Ganoderma lucidum* as determined by cupric reducing antioxidant capacity (CUPRAC).

Aqueous extract of *Ganoderma lucidum *(mg/mL)	Absorbance values at 450 nm
0.10	0.15 ± 0.01^a^
0.25	0.37 ± 0.01^b^
0.50	0.75 ± 0.03^c^
0.75	1.10 ± 0.03^d^
1.00	1.42 ± 0.03^e^
1.50	1.94 ± 0.03^f^
Ascorbic acid (5 × 10^−3^ g/mL)	1.89 ± 0.03^f^

Data are expressed as mean ± S.E.M (*n* = 3).

Means with different superscripts were significantly different (*P* < 0.05).

**Table 3:** The effects of aqueous extract of *G. lucidum* on wound closure in streptozotocin-induced diabetic rats.

Treatment	Day 8		Day 12	
	Wound closure (mm^2^)	Percentage of wound closure (%)	Wound closure (mm^2^)	Percentage of wound closure (%)
Aqueous cream (negative control)	39.23 ± 6.1^a^	11	155.81 ± 20.6^d^	62
Intrasite gel	136.86 ± 22.8^b^	55	175.68 ± 23.1^cd^	70
Aqueous cream containing 5% extract of *G. lucidum *	120.98 ± 17.3^b^	47	217.12 ± 30.1^c^	83
Aqueous cream containing 10% extract of *G. lucidum *	149.12 ± 16.5^c^	60	242.08 ± 7.8^a^	97
Aqueous cream containing 15% extract of *G. lucidum *	110.76 ± 16.0^d^	44	213.90 ± 16.1^bc^	86
Aqueous cream containing 20% extract of *G. lucidum *	101.04 ± 23.1^d^	40	226.62 ± 13.5^b^	91

Data are expressed as mean ± S.E.M (*n* = 6). Means with different superscripts were significantly different (*P* < 0.05).

**Table 4:** *In vivo* antioxidant activity and oxidative status of rats during wound healing on day 7 after operation.

Treatment	CUPRAC (A_450_)	AOPP (*μ*mol/L chloramine-T equivalents)	LPO (nM of TEP equivalents)
Aqueous cream (negative control)	0.19 ± 0.03^a^	627.3 ± 32.6^a^	22.30 ± 1.60^a^
Intrasite gel	0.16 ± 0.01^a^	581.3 ± 71.9^a^	19.99 ± 2.18^a^
Aqueous cream containing 5% extract of *G. lucidum *	0.20 ± 0.01^a^	484.0 ± 67.3^ab^	20.91 ± 0.47^a^
Aqueous cream containing 10% extract of *G. lucidum *	0.23 ± 0.04^ab^	253.5 ± 38.5^c^	18.46 ± 0.84^a^
Aqueous cream containing 15% extract of *G. lucidum *	0.30 ± 0.06^b^	373.0 ± 58.1^bc^	20.52 ± 1.27^a^
Aqueous cream containing 20% extract of *G. lucidum *	0.23 ± 0.01^ab^	353.8 ± 72.0^bc^	20.02 ± 2.04^a^

Data are expressed as mean ± SD (*n* = 6). Means with different superscripts were significantly different (*P* < 0.05).
